# Alterations in blood pressure dependent activation of leg muscles during standing following bed rest mimic those observed with ageing

**DOI:** 10.3389/fphys.2025.1426648

**Published:** 2025-04-29

**Authors:** Malcom Tremblay, Da Xu, Ajay K. Verma, Nandu Goswami, Andrew P. Blaber

**Affiliations:** ^1^ Department of Biomedical Physiology and Kinesiology, Simon Fraser University, Burnaby, BC, Canada; ^2^ Department of Neurology, University of Minnesota, Minneapolis, MN, United States; ^3^ Division of Physiology and Pathophysiology, Otto Löwi Research Center for Vascular Biology, Immunity and Inflammation, Medical University of Graz, Graz, Austria; ^4^ College of Medicine, Mohammed Bin Rashid University of Medicine and Health Sciences, Dubai, United Arab Emirates

**Keywords:** ageing, bed rest, baroreflex, muscle-pump, orthostatic hypotension

## Abstract

**Introduction:**

Baroreflex-mediated activation of lower leg muscles (i.e., muscle-pump baroreflex) has been suggested to play a significant role in blood pressure regulation during standing. Compromised muscle-pump baroreflex because of ageing or prolonged inactivity could contribute to orthostatic hypotension. Understanding the contribution of individual lower leg muscles to blood pressure regulation could lead to the development of muscle-specific strategies to prevent orthostatic hypotension associated with muscle-pump baroreflex dysfunctions.

**Methods:**

In this study, individual muscle (tibialis anterior (TA), lateral soleus (SOL), medial gastrocnemius (MG), and lateral gastrocnemius (LG)) responses to blood pressure changes upon a supine-to-stand orthostatic challenge were examined in young adult male participants (35 ± 2 years) before and after 60 days of 6° head-down tilt bed rest (HDBR). By analyzing the interactions between systolic blood pressure (SBP) and heartbeat-by-heartbeat electromyogram impulse (EMG_imp_) during standing, the interactions between SBP and EMGimp including muscle-pump baroreflex were characterized by fraction time active (FTA) and response gain from wavelet transform coherence analysis and the causality values using convergent cross mapping method for individual leg muscles. Since inactivity and ageing are common causes of orthostatic intolerance, the HDBR results were compared with those from young and older individuals in a previously published study to investigate the similarities in their effects on muscle-pump baroreflex.

**Results:**

During standing, FTA reduced for all muscles except MG following HDBR and was lower in older compared to younger participants. Muscle-pump baroreflex causality (SBP→EMG_imp_) reduced for all muscles following HDBR and was lower for LG and SOL muscles in older compared to younger adults. The mechanical muscle-pump causality (SBP→EMG_imp_) was not affected by HDBR or by age. Increased TA muscle-pump baroreflex gain post-HDBR may point to a compensatory mechanism for decreased active control.

**Conclusions:**

Our results showed striking similarities in the alteration of muscle-pump baroreflex induced via ageing and HDBR, suggesting strong commonalities between ageing and long-term inactivity in terms of the adverse effects on baroreflex mediated control of lower leg muscle activities in response to orthostatic challenge.

## 1 Introduction

Orthostatic hypotension, a physiological condition where blood pressure drops abruptly during an upright stance, often occurs in older persons (Eigenbrodt et al., 2000; Goswami et al., 2017; Ricci et al., 2015), in persons following long-lasting bed rest confinements (Barbic et al., 2019; Convertino et al., 1990; Kamiya et al., 2003), and in astronauts after returning to Earth (Blaber et al., 2013; Buckey et al., 1996; Meck et al., 2004). Upon standing, blood pools in the lower body, and the resultant drop in blood pressure is detected by baroreceptors to activate baroreflex responses to regulate blood pressure. One of such regulatory mechanism that has been largely investigated is the arterial baroreflex, where blood pressure alters baroreceptor output and hence heart rate and vascular resistance (Blaber et al., 1995; Jacobsen et al., 1993; Rowell, 1993; Victor and Mark, 1985). While standing, skeletal muscle contractions in the legs also play an important role in blood pressure regulation by propelling pooled blood in the veins back to the heart (mechanical muscle-pump). Considering muscle contractions through the neuromuscular pathway, we proposed a neurally-mediated mechanism of muscle-pump, in which decreased blood pressure activates reflex responses including skeletal muscle-pump activity through a hypothesized cardio-postural control center (Blaber et al., 2009; Garg et al., 2013; 2014). We named this feed-back reflex mechanism muscle-pump baroreflex, which forms a closed-loop system with the mechanical muscle-pump. Our research has shown that the muscle-pump baroreflex could play an important role in blood pressure regulation during standing (Verma et al., 2017; Xu et al., 2017).

Impaired muscle-pump baroreflex and unaltered mechanical muscle-pump have been observed in healthy young subjects after 60 days of head-down bed rest (HDBR), indicating a possible link between reduced reflex responses and orthostatic intolerance after HDBR (Xu et al., 2020). While the aggregate muscle activity could describe the changes in muscle-pump baroreflex with HDBR (Xu et al., 2020), a previous investigation (Verma et al., 2019) showed that individual leg muscles contribute differently to the muscle-pump baroreflex response during standing and with age. To what extent 60-day HDBR, as an effective model to simulate microgravity (Harris et al., 2022), affects the muscle-pump baroreflex of individual leg muscles, remains unclear.

This research sought to understand the fundamental changes in the muscle-pump baroreflex mechanism of the individual leg muscles following prolonged HDBR. Given the commonalities between spaceflight, inactivity, and ageing (Goswami, 2017), we aimed to compare bed rest associated changes (pre- vs. post-HDBR) in the muscle-pump baroreflex of individual leg muscles with our previously published results in this phenomenon in young and older participants (Verma et al., 2019). This quantitative comparison could aid in the development of muscle-specific strategies targeted towards preventing the dysfunction of the lower leg muscle-pump baroreflex which could have far-reaching clinical implication in preventing episodes of dizziness in returning astronauts, people confined to bed for prolonged periods, and older individuals. We hypothesized prolonged HDBR would have a negative impact on the muscle-pump baroreflex of the individual leg muscles and such HDBR-induced impairment would mimic that observed with ageing.

## 2 Materials and methods

### 2.1 Protocols

The experiments were conducted at the *Institut de Médecine et de Physiologie Spatiales* (MEDES), a *Centre National d’Études Spatiales* (CNES) operated facility in Toulouse, France, as part of an ESA funded prolonged HDBR study. Two HDBR campaigns were included with participation of 10 male volunteers each campaign subjected to HDBR at 6° for 60 days. Ethical approval for all research was received from the *Comité de Protection des Personnes/CPP Sud-Ouest Outre-Mer I* and the *Agence Française de Sécurité Sanitaire des Produits de Santé* for each aspect of the study and scientific protocols. Research associated with our study was approved by the Office of Research Ethics at Simon Fraser University. A written informed consent was signed by each participant. Research was conducted in compliance with the guidelines and regulations of the above agencies. Detailed participant selection criteria were agreed upon by MEDES and the 16 scientific teams involved and were described by Xu et al. (2020).

In this paper, we examine the individual muscles that were combined in the results presented by Xu et al. (2020) where a detailed research protocol has been described. Briefly, a supine-to-stand (StS) test was used to evoke the muscle-pump baroreflex and was performed in the morning during baseline data collection (BDC) 12 days before (BDC12) and 2 days before (BDC02) HDBR, immediately after HDBR (Recovery Day 0: R+0) and 8 days after (R8). The StS test on BDC02 and R+0 was performed 45 min after the ESA bed rest core data syncope test which included a head-up tilt (HUT) plus graded lower body negative pressure (LBNP) sequence to pre-syncope (Goswami et al., 2012a; Grasser et al., 2009; Hinghofer-Szalkay et al., 2011). The StS test was composed of a 5-min eyes closed quiet supine followed by a 6-min eyes closed quiet stand. During the quiet stand, the participants were positioned at the center of a force platform with their feet placed parallel and 5 cm apart.

### 2.2 Data collection

During the StS test, a standard Lead II electrode configuration was used to record bipolar three-lead electrocardiography (ECG) (FD-13, Fukuda Denshi Co. Ltd., Tokyo, Japan). Continuous blood pressure (BP) was acquired with a non-invasive Portapres (FMS, Amsterdam, Netherlands) with absolute blood pressure height-corrected to heart level. Transdermal differential recording of surface electromyography (EMG) was performed using the Bagnoli-8 (Delsys Inc., MA, United States) EMG system from four bilateral lower leg muscles of both legs: tibialis anterior (TA), lateral soleus (SOL), medial gastrocnemius (MG), and lateral gastrocnemius (LG). The sites for EMG sensor placement were chosen based on recommendations from the SENIAM project (Hermens et al., 1999). Data were acquired at a sampling rate of 1,000 Hz through a National Instruments USB-6218 16-bit data acquisition platform and Labview 2013 software (National Instruments Inc., TX, United States).

### 2.3 Data analysis

**FIGURE 1:**
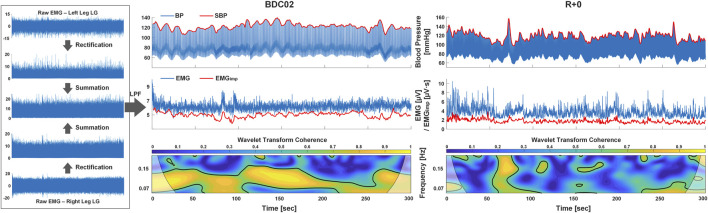
Example of the wavelet transform coherence (WTC) analysis between systolic blood pressure (SBP) and electromyogram impulse (EMG_imp_) from one participant on BDC02 and R+0. Beat-by-beat SBP and EMG_imp_ (red solid line) were extracted from the continuous blood pressure and EMG signals (blue solid line), where the EMG signal was low-pass filtered (LPF) from the summation of rectified individual muscle EMG from both legs (left panel). SBP and EMG_imp_ were then re-sampled to 10 Hz for the calculation of time-frequency distribution of wavelet transform coherence between them. The area of significant coherence above the threshold (indicated by the bold contour on the time-frequency distribution) were identified and divided by the total area of the low frequency (LF, 0.07–0.15 Hz) band to calculate the fraction time active (FTA).

Data analyses were performed in MATLAB (MathWorks, MA, United States) from the last 5 minutes of the 6-min quiet standing data (Figure 1). Heart rate (HR) and beat-by-beat systolic blood pressure (SBP) was identified based on QRS complex detection from ECG by the Pan-Tompkins algorithm (Pan and Tompkins, 1985; Xu et al., 2017) and the continuous BP waveform. Individual EMG from MG, LG, TA, and SOL were determined by adding the rectified EMG signals from both legs and a fourth order Butterworth low-pass filter with cut-off frequency of 5 Hz applied. The EMG impulse (EMG_imp_) of each muscle was then calculated as the area under the EMG envelope within each heartbeat to represent the muscle contraction strength on a heartbeat-by-heartbeat basis (Xu et al., 2017). EMG impulse was used because, from a beat-by-beat perspective, it represents the contribution of motor-neuron output on the muscle-pump to cardiac filling within a heartbeat (and hence blood pressure in the next beat) as it is collectively affected by both EMG activities and the heart period, i.e., the accumulated effects of muscle-pump over each heartbeat. That is, a brief strong contraction can be equivalent to weaker contractions over a longer period and contraction of the same magnitude would produce higher overall strength over longer heartbeats. Since cardiovascular data were processed and analyzed on a beat-by-beat basis, EMG_imp_ is more appropriate to assess the interactions between blood pressure and muscle-pump activities. Following the beat-by-beat alignment of cardiovascular and muscle-pump data, all beat-by-beat time series were re-sampled to 10 Hz using spline interpolation prior to further analysis.

Wavelet transform coherence (WTC) analysis and convergent cross mapping (CCM) causality methods were used to characterize the interaction and causal relationship between SBP and EMG_imp_ for each muscle. In the WTC analysis (Figure 1), the Morlet wavelet was used to provide wavelet transform coherence distributions (Garg et al., 2013; 2014) to represent the interactions between SBP and EMG_imp_ in both time and frequency domains. The Monte Carlo method was used to extract the threshold of significant coherence (Grinsted et al., 2004; Xu et al., 2020). The muscle-pump baroreflex was investigated in the low-frequency range (LF, 0.07–0.15 Hz) based on our previous work (Garg et al., 2014; Xu et al., 2020). To represent the average time of active interaction within the LF range, the fraction time active (FTA) was computed as the area of WTC distribution where coherence values were above the significant coherence threshold in the LF band divided by the total area of the LF band. The muscle-pump baroreflex response gain (SBP→EMG_imp_) was calculated as the cross wavelet transform of SBP and EMG_imp_ divided by the magnitude squared of the wavelet transform of SBP (Grinsted et al., 2004; Xu et al., 2020) and averaged over regions of significant WTC in LF band.

The bidirectional causalities between SBP and EMG_imp_ were calculated using the CCM method (Sugihara et al., 2012). Causality provides a quantitative indicator of the cause and effect relationship between two signals with values ranging from 0 (absence of causality) to 1 (maximum causality). Causalities on both directions were assessed: mechanical effect of muscle-pump activity on blood pressure (EMG_imp_→SBP) and neurally mediated activation of muscle-pump in response to blood pressure changes (SBP→EMG_imp_). Detailed mathematical representation of the CCM method can be found in our previous work (Verma et al., 2017) and the supplementary material of (Sugihara et al., 2012).

Statistical analysis was performed with JMP 15 (SAS Institute). The two-way repeated measures ANOVA was modelled for within-participant analysis to compare the main effects of the day of the study (Test Day) and the lower leg muscles (Muscle) selected in this study. Significance over main effects and interactions was assessed with Tukey’s HSD (*post hoc*) test. Significance was defined as α < 0.05.

## 3 Results

### 3.1 Participants and physiological measures

We analyzed the data from the 19 male participants reported by Xu et al. (Xu et al., 2020) where one participant was excluded due to non-compliance with bed rest and study rules. The participants had a mean age of 35 ± 2 years, a mean height of 176 ± 1 cm, and a mean weight of 72.9 ± 1.7 kg; mean ± SEM. Compared to pre-HDBR, their weight was on average lighter on R+0 (72.2 ± 1.6 kg, p < 0.01) and recovered by R8 (73.1 ± 1.6 kg). Calf circumference was significantly reduced from pre-HDBR (36.5 ± 0.5 cm) to R+0 (34.1 ± 0.5 cm, p < 0.01) with recovery on R8 (36.1 ± 0.5 cm). During quiet stand, higher HR (129 ± 3 bpm) and lower SBP (123 ± 4 mmHg) were observed on R+0 compared to BDC12 (HR: 84 ± 3 bpm, SBP: 138 ± 4 mmHg). On R8, while SBP recovered to pre-HDBR level (128 ± 4 mmHg), HR remained elevated (96 ± 3 bpm). Aggregate EMG activity and EMG_imp_ from all muscle groups were significantly attenuated after HDBR (EMG: 79.2 ± 7.7 µV on R+0 and 64.4 ± 7.7 µV on R8; EMG_imp_: 38.5 ± 5.6 μV s on R+0 and 41.9 ± 5.6 μV s on R8) compared to pre-HDBR levels (EMG: 82.7 ± 7.7 µV on BDC12 and 86.8 ± 7.7 µV on BDC02; EMG_imp_: 60.2 ± 5.6 μV s on BDC12 and 64.6 ± 5.6 μV s on BDC02).

### 3.2 Wavelet transform coherence analysis

**FIGURE 2:**
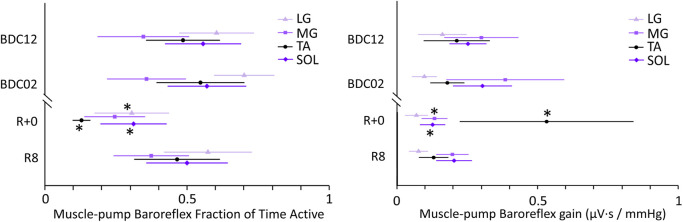
Muscle-pump baroreflex fraction time active (left panel) and gain (right panel) in the low frequency band obtained from individual leg muscles (tibialis anterior (TA), lateral soleus (SOL), medial gastrocnemius (MG), and lateral gastrocnemius (LG)) before and after head-down bed rest. Muscle data in shaded violet represent muscles predominantly associated with medio-lateral sway, and those in black anterior-posterior sway. Means ± 95% confidence interval are represented. *: significantly different from BDC02 and BDC12.

Overall, FTA pooled from all the muscles was significantly reduced post-HDBR on R+0 compared to BDC12 (p < 0.0001) and BDC02 (p < 0.0001) and had recovered by R8 (p < 0.0001 compared to R+0) (Table 1). In terms of overall activity by muscle group, FTA across all test days was higher in the LG compared to the TA (p < 0.0001) and MG (p = 0.004). The FTA of SOL was also greater than the MG (p < 0.0001). Analysis of Test Day by Muscle interactions showed that all but the FTA of the MG, which had the lowest overall FTA (Table 1), were reduced on R+0 compared to BDC12 and BDC02 (Figure 2).

**TABLE 1:** Results of wavelet transform coherence and causality analysis from two-way repeated measures Analysis of Variance (Test Day and Muscle group).

	Wavelet transform coherence		Causality	
	FTA	Gain (SBP→EMG_imp_) (µV s/mmHg)	SBP→EMG_imp_	EMG_imp_→SBP
Muscle				
LG	0.55 ± 0.05	0.10 ± 0.03	0.85 ± 0.01	0.92 ± 0.01
MG	0.33 ± 0.05	0.26 ± 0.03	0.80 ± 0.01	0.90 ± 0.01
SOL	0.49 ± 0.05	0.22 ± 0.03	0.83 ± 0.01	0.91 ± 0.01
TA	0.41 ± 0.05	0.26 ± 0.03	0.83 ± 0.01	0.91 ± 0.01
p	<0.0001	<0.0001	0.008	0.062
Test day				
BDC12	0.51 ± 0.05	0.23 ± 0.03	0.87 ± 0.01	0.91 ± 0.01
BDC02	0.54 ± 0.05	0.24 ± 0.03	0.89 ± 0.01	0.92 ± 0.01
R+0	0.25 ± 0.05	0.22 ± 0.03	0.73 ± 0.01	0.91 ± 0.01
R8	0.49 ± 0.05	0.15 ± 0.03	0.83 ± 0.01	0.91 ± 0.01
p	<0.0001	0.0903	<0.0001	0.348
Test day * Muscle				
p	0.0926	<0.0001	0.0912	0.905

FTA, fraction time active; Gain, muscle-pump baroreflex gain; LG, lateral gastrocnemius; MG, medial gastrocnemius; SOL, soleus; TA, tibialis anterior; SBP, systolic blood pressure; EMG_imp_, electromyogram impulse. P values indicate the significance level of the main effects (Test day and Muscle) and the interaction term (Test day * Muscle).

No significant main effects were observed in the muscle-pump baroreflex gain after HDBR (p = 0.0903, Table 1). However, a significant main effect for individual muscle indicated that LG gain was lower than other muscles (p < 0.0001, Table 1). Analysis of Test Day by Muscle interactions revealed the TA as the only muscle to show an increase in gain at R+0 compared to other test days (p ≤ 0.005) and other muscles (p < 0.0001), while the gain of the MG and the SOL decreased with the LG unchanged post-HDBR (Figure 2).

### 3.3 Causality analysis

**FIGURE 3:**
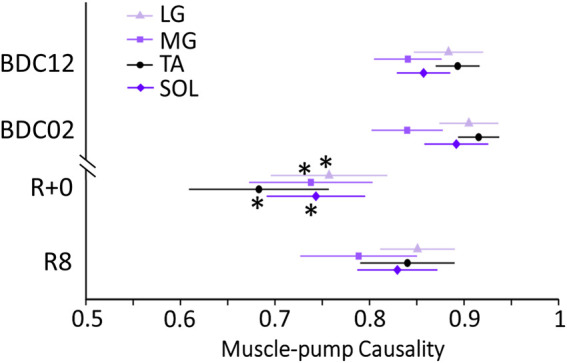
Muscle-pump baroreflex causalities (SBP→EMG_imp_) for individual leg muscles (tibialis anterior (TA), lateral soleus (SOL), medial gastrocnemius (MG), and lateral gastrocnemius (LG)) before and after head-down bed rest (HDBR). Muscle data in shaded violet represent muscles predominantly associated with medio-lateral sway, and those in black anterior-posterior sway. *: significantly different from BDC02 and BDC12.

Causality of the muscle-pump baroreflex (SBP→EMG_imp_) had significant main effects for Test Day (Table 1; Figure 3). All muscle groups’ baroreflex causality dropped on R+0 compared to BDC12 (p < 0.0001) and BDC02 (p < 0.0001) (Table 1). By R8, muscle-pump baroreflex causality was increased from R+0 (p < 0.0001) but not recovered to BDC02 (p < 0.0001) or BDC12 (p = 0.016) values (Table 1). There was also a significant main effect for muscles (p = 0.0008) in that muscle-pump baroreflex causality for the LG was consistently higher than the MG (p = 0.004) (Table 1).

No changes were observed in the non-baroreflex direction (EMG_imp_→SBP) causality (Table 1) over all days and across all muscle groups (p = 0.91).

### 3.4 Comparison with ageing

Significant changes in FTA, gain, and causality from baseline (BDC02) and the first day after HDBR (R+0) (Table 2) were compared to the differences between the young (24 ± 2 years, n = 18, 10 males) and older participants (69 ± 4 years, n = 14, six males) published in our previous paper (Verma et al., 2019). Commonalities were observed with FTA and causality. When all the participants in the ageing study were included (males and females), FTA of LG, TA, and SOL muscles was reduced with age and bed rest. A change in the FTA of the MG was observed with age, but not with HDBR. Bed rest produced a significant decline in the muscle-pump baroreflex causality of all the muscle groups, while only those of LG and SOL declined with age. Like ageing, there was no change in the causality of muscle-pump mechanics (EMG_imp_→SBP). The changes in muscle-pump baroreflex gain seen with bed rest were not observed with ageing.

**TABLE 2:** Comparison of changes in muscle-pump baroreflex characteristics with age (Verma et al., 2019) (young: n = 18, 10 males; older: n = 14, six males) and bed rest (19 males) on BDC02 and R+0.

		LG	MG	SOL	TA
FTA, %SC	Age (All)	↓	↓	↓	↓
	Age (male only)	↓	↔	↓	↔
	Bed rest	↓	↔	↓	↓
Gain (SBP→EMG_imp_)	Age (All)	↔	↔	↔	↔
	Age (male only)	↔	↔	↔	↔
	Bed rest	↔	↓	↓	↑
Causality (SBP→EMG_imp_)	Age (All)	↓	↔	↓	↔
	Age (male only)	↔	↔	↔	↔
	Bed rest	↓	↓	↓	↓
Causality (EMG_imp_→SBP)	Age (All)	↔	↔	↔	↔
	Age (male only)	↔	↔	↔	↔
	Bed rest	↔	↔	↔	↔

FTA, fraction time active; %SC, percent significant coherence (Verma et al., 2019), equivalent to FTA; Gain, muscle-pump baroreflex gain; LG, lateral gastrocnemius; MG, medial gastrocnemius; SOL, soleus; TA, tibialis anterior. The arrows indicate a significant change with age or bed rest in a muscle: ↓: decrease; ↑: increase; ↔: no change.

Causality, FTA, and gain were not significantly different between the BDC02 (pre-bed rest) and the young participants in the Verma et al. study (Verma et al., 2019) when compared to all young participants or just the young male participants. Differences were observed between older participants and post-bed rest on R+0 (Table 3). In instances where no change was observed with ageing, but was with HDBR (Table 2), there was a significant difference between bed rest R+0 and older participants. The LG muscle-pump baroreflex causality and SOL gain for older males was higher than seen following HDBR. Muscle-pump baroreflex causality of TA in all older participants and with males only was significantly greater than the post-HDBR value.

**TABLE 3:** Comparison of changes in muscle-pump baroreflex characteristics from older participants (Verma et al., 2019) (n = 14, six males) and bed rest on R+0 (19 males).

		LG	MG	SOL	TA
FTA, %SC	Older (all)	0.21 (0.03, 0.28)	0.14 (0.02, 0.20)	0.15 (0.03, 0.31)	0.20 (0.04, 0.50)
	Older (males)	0.14 (0.02, 0.25)	0.12 (0.02, 0.20)	0.10 (0.02, 0.22)	0.14 (0.09, 0.20)
	Bed rest	0.30 (0.16, 0.44)	0.24 (0.13, 0.35)	0.31 (0.19, 0.43)	0.13 (0.01, 0.16)
Gain (SBP→EMG_imp_)	Older (all)	0.14 (0.04, 0.42)	0.21 (0.05, 1.21)	0.25 (0.04, 0.70)	0.98 (0.04, 2.48)
	Older (males)	0.03 (0.00, 0.07)	0.35 (0.10, 0.69)	0.33 (0.17, 0.50)*	0.93 (0.05, 2.67)
	Bed rest	0.06 (0.01, 0.11)	0.13 (0.08, 0.18)	0.12 (0.07, 0.17)	0.53 (0.22, 0.84)
Causality (SBP→EMG_imp_)	Older (all)	0.81 (0.69, 0.94)	0.80 (0.63, 0.94)	0.79 (0.67, 0.94)	0.87 (0.79, 0.96)*
	Older (males)	0.85 (0.73, 0.95)*	0.83 (0.56, 0.94)	0.79 (0.53, 0.95)	0.90 (0.82, 0.97)*
	Bed rest	0.76 (0.71, 0.81)	0.73 (0.67, 0.79)	0.74 (0.69, 0.79)	0.68 (0.60, 0.76)

FTA, fraction time active; %SC, percent significant coherence (Verma et al., 2019) was converted to FTA (%SC divided by 100). Gain, muscle-pump baroreflex gain; LG, lateral gastrocnemius; MG, medial gastrocnemius; SOL, soleus; TA, tibialis anterior. Values are shown as: mean (5%, 95% confidence limits). *: value is different from bed rest.

## 4 Discussion

Maintenance of upright stance is an integrative process involving postural stability and prevention of orthostatic hypotension. In this work, the lower leg muscles associated with both systems were examined for their contribution to blood pressure regulation following 60 days of HDBR and inactivity. Significant post-HDBR declines were revealed in a) activation (FTA) of the LG, TA, and SOL; b) baroreflex gain of the MG and SOL; and c) causality of all four muscle groups. When compared with differences between young and older adults, HDBR changes in LG characteristics matched, however, the other three muscle groups exhibited some, but not all, of these behaviors. Notably, MG FTA declined, and gain did not change with age, while MG FTA did not change and gain decreased with HDBR, and TA gain remained unchanged with age and increased with HDBR. The data provided a unique opportunity to examine skeletal muscle-pump baroreflex adaptations with active ageing and bed rest immobilization.

### 4.1 HDBR and muscle-pump baroreflex

Lower leg muscles have been shown to engage with blood pressure fluctuations during standing to maintain blood pressure homeostasis (Magder, 1995; Masterson et al., 2006; Smit et al., 1999; Ten Harkel et al., 1994; van Dijk et al., 2005). In this study, significant impairment of muscle-pump baroreflex (SBP→EMG_imp_) causality was observed in all muscles. However, the changes in FTA and gain were more varied. Interaction between blood pressure and skeletal muscle activity (FTA) was reduced in three of the muscle groups (LG, SOL, TA) with no change in the MG, while the gain remained unchanged in the LG, reduced in the MG and SOL, and increased in the TA. The SOL and gastrocnemius muscles show a net reduction in muscle-pump baroreflex control; however, the TA did not.

We hypothesized that the muscle-pump baroreflex of all four muscles would be compromised after HDBR, since exercise was strictly prohibited. Muscle wasting was severe, especially in the lower extremities, as previously reported with calf circumference measurements (Xu et al., 2020). As well, the aggregate muscle data showed significant reductions in FTA, baroreflex gain, and causality (Xu et al., 2020). With these observations, the increase in the gain of the TA was unexpected, although the changes in FTA and causality were in the predicted direction. This may indicate that the TA shows a possible compensatory effect with an increase in muscle-pump baroreflex gain as an offset to decreased activation time and causality (Figure 2).

Given the differential changes in FTA and muscle-pump baroreflex gain between the muscle groups, these data suggest that muscle-pump motor control adaptations are independent of global reductions in baroreflex coupling. These muscles serve a dual purpose with remaining upright: postural stability and blood pressure regulation. The variation in responses may result from the interaction between the two modalities. Xu et al. (2020) reported a doubling of the mean deviation of the center of pressure (COPr) and the mean velocity of movement (COPr_v_) of these participants on R+0 compared to baseline. There was a decrease in postural stability in the participants, which would cause greater compensatory output from the muscles. Although the data quoted (Xu et al., 2020) were for overall sway, directionality is partially differentiated between muscle groups. Of the muscles measured in this study, the LG, MG target medio-lateral sway, whereas the TA and SOL are predominantly in anterior-posterior sway. In this context, integrated cardio-postural events (Garg et al., 2014) may explain variations in baroreflex. As a group, the medio-lateral control muscles showed balance, with only one experiencing a decline in FTA or gain. With the anterior-posterior control muscles, the SOL had significant impairment in all aspects of the muscle-pump baroreflex, while the TA, in opposition, had a significant increase in gain. The causal relationship between muscle pump mechanics and blood pressure (EMG_imp_→SBP) after 60-day HDBR remained intact (Table 1). Given the magnitude of change in postural sway, it is probable that postural activation of the TA contained significant overlap with variations in blood pressure, which added to the gain (Garg et al., 2014).

### 4.2 HDBR and ageing comparisons

While HDBR has been long recognized as an effective model to simulate microgravity (Goswami, 2017; Goswami et al., 2015), recent research has suggested that bed rest is also a suitable model to study muscle decline on ageing during inactivity (Di Girolamo et al., 2021). In our previous study (Verma et al., 2019) cardio-postural interactions of the same four lower leg muscles (MG, LG, TA, and SOL) as this study were examined in young (24 ± 2 years, n = 18, 10 males) and older (69 ± 4 years, n = 14, six males) participants. The present study was composed of all males (35 ± 2 years, n = 19) who experienced 60 days of inactivity in HDBR.

The main findings from the work of Verma et al. was that FTA and muscle-pump baroreflex causality were lower in older compared to young participants, while the baroreflex response gain (SBP→EMG_imp_) and the mechanical muscle-pump causality (EMG_imp_→SBP) remained unchanged (Verma et al., 2019). These are in accordance with our previous combined analysis of cardio-postural changes following HDBR in the same participants as in this study (Xu et al., 2020). Similarities are also found in the current analysis of individual muscles in terms of FTA and causality (Table 2), which suggests that ageing and prolonged HDBR induce similar degradation in muscle-pump baroreflex. They provide compelling support for HDBR as a model for improving our understandings of ageing associated changes in cardiovascular and postural systems. These understandings can provide a rationale for devising effective countermeasures to mitigate the negative impact of and/or prevent falls related to dysfunction in the baroreflex control of blood pressure upon standing.

In fact, the correspondences in the impairment of muscle-pump baroreflex from ageing and HDBR (Table 2) are striking. The pattern in the distribution of FTA across muscle groups was analogous when comparing the percent significant coherence (equivalent to FTA converted to percentage) between the young group with pre-HDBR and older group with post-HDBR. This shows possible common effects of ageing and prolonged inactivity on neurally mediated mechanisms for blood pressure control during standing.

FTA was reduced in the LG, TA, and SOL with HDBR (Figure 2) and ageing (Verma et al., 2019). However, unlike with ageing, no change in FTA was observed with the MG after HDBR (Table 2). Indeed, overall lower FTA and muscle-pump baroreflex causality in the MG were observed compared with other muscles (Table 1; Figures 2, 3), signifying a low association between MG and muscle-pump baroreflex. Similarly, our results from young and older groups suggested less reliance on the MG for the muscle-pump baroreflex (Verma et al., 2019).

The TA muscle showed a distinct pattern with a significant increase in muscle-pump baroreflex gain post-HDBR (Figure 2). In the ageing data (Verma et al., 2019), the EMG_imp_ of TA muscle was also reported to be higher in older persons. LG and SOL in both the ageing and HDBR data showed a general decrease in FTA and muscle-pump baroreflex causality (Table 2), signalling an effective decline in muscle-pump baroreflex control. These data suggest differential changes in central and/or peripheral motor control regarding the different muscle groups and that these changes were similar between HDBR and ageing. Further research into mechanisms is warranted since the changes noted by Verma et al. (2019) were in older persons who had maintained physical fitness.

### 4.3 Limitations and future work

Although we used the same raw data as the previously published report by Xu et al. (2020), slight differences exist in these results because of segregation into individual muscle signals prior to WTC and CCM analyzes compared to the use of aggregate EMG (Xu et al., 2020). This may also explain the larger differences in baroreflex gain between the two studies.

The current study was composed of all male participants. However, there is a growing body of evidence to suggest that women demonstrate a lower tolerance for orthostatic stress (Schondorf and Low, 1992). While studies have shown sex differences in arterial baroreflex responses to orthostatic challenge (Arzeno et al., 2013; Convertino, 1998), the sex effects on muscle-pump baroreflex in response to blood pressure changes and prolonged bed rest is yet to be understood. Therefore, future studies including both males and females are warranted (Shankhwar et al., 2023).

Despite the validation through physiological modeling and analysis methods, the proposed muscle-pump baroreflex is based on a hypothesized cardio-postural control center, which is a black-box in the model. The actual origin of the neuromuscular activities in response to blood pressure changes is unclear. Our future work could include animal experiments or additional brain imaging/neuromuscular imaging (e.g., MRI) on human participant to obtain further evidence of muscle-pump baroreflex during standing.

EMG impulse (EMG_imp_) was used in this study to take into account the accumulated effects of motor-neuron input and the muscle-pump on cardiac filling over each heartbeat. While beat-by-beat averaged EMG (EMG_ave_) also represents the muscle-pump activities, the causality analysis between SBP and EMG_ave_ revealed similar changes of muscle-pump baroreflex causality across Test Day compared with EMG_imp_, but the differences across Muscle observed with EMG_imp_ were absent (Tables 1, 4). If changes in SBP affect HR and muscle-pump activities independently, the causality results obtained from EMG_imp_ would show no significant difference across Muscle as with EMG_ave_. These results indicate that, rather than a simple modulation of heart period on EMG_ave_, EMG_imp_ may be able to shed more insights into the effects of blood pressure changes on different lower leg muscles (e.g., interactions between arterial baroreflex and muscle-pump baroreflex). This is also aligned with our hypothesis of the cardio-postural control center where the arterial and muscle-pump baroreflexes are likely interacted with each other. Therefore, it is reasonable to hypothesize that EMG_imp_ provides additional useful information in the assessment of muscle-pump baroreflex. Nevertheless, such hypothesis requires further validation and future comparison studies on different forms of EMG data used in the analysis would be necessary.

**TABLE 4:** Results of causality analysis from two-way repeated measures analysis of variance (Test day and Muscle group) using beat-by-beat averaged EMG (EMG_ave_).

Causality		
	SBP→EMG_ave_	EMG_ave_→SBP
Muscle		
LG	0.74 ± 0.02	0.90 ± 0.01
MG	0.74 ± 0.02	0.89 ± 0.01
SOL	0.74 ± 0.02	0.89 ± 0.01
TA	0.75 ± 0.02	0.89 ± 0.01
p	0.918	0.786
Test day		
BDC12	0.79 ± 0.02	0.89 ± 0.01
BDC02	0.79 ± 0.02	0.89 ± 0.01
R+0	0.67 ± 0.02	0.90 ± 0.01
R8	0.73 ± 0.02	0.89 ± 0.01
p	<0.0001	0.13
Test day * Muscle		
p	0.846	0.839

LG, lateral gastrocnemius; MG, medial gastrocnemius; SOL, soleus; TA, tibialis anterior; SBP, systolic blood pressure; EMG_ave_, averaged electromyogram within each heartbeat. P values indicate the significance level of the main effects (Test day and Muscle) and the interaction term (Test day * Muscle).

## 5 Conclusion

Muscle-pump baroreflex, a regulatory mechanism which facilitates skeletal muscle activation in response to changes in blood pressure during standing, was suggested to play a significant role in blood pressure regulation upon orthostatic challenge (Goswami et al., 2012b). The participants in this study displayed a significant post-HDBR reduction in muscle-pump baroreflex. This underscores the dysfunction in critical blood pressure regulatory mechanisms induced by prolonged HDBR, which could explain the frequent episodes of dizziness and/or fall in people experienced prolonged bed rest and astronauts upon return to Earth. Strong commonalities between ageing and prolonged bed rest were found in terms of their adverse effects on muscle-pump baroreflex through the interaction time (FTA) and the control causality. While the overall results are in accordance with our previous study based on the aggregate muscle data (Xu et al., 2020), analysis on individual muscles revealed differential changes between the muscle groups which suggested a lower association of the muscle-pump baroreflex with the MG muscle and a possible compensatory effect from the TA muscle against the post-HDBR impairment of muscle-pump baroreflex and postural stability. Clinically, our findings will have implications in the development of muscle-specific strategies to counter the adverse effect of long-term inactivity, ageing, and spaceflight on muscle-pump baroreflex.

## Data Availability

The raw data supporting the conclusion of this article will be made available by the authors, without undue reservation.
